# Magnetic resonance imaging prognostic factors for survival and relapse in dogs with meningoencephalitis of unknown origin

**DOI:** 10.3389/fvets.2024.1370882

**Published:** 2024-02-28

**Authors:** Rita Gonçalves, Steven De Decker, Gemma Walmsley, Thomas W. Maddox

**Affiliations:** ^1^Department of Veterinary Science, Small Animal Teaching Hospital, University of Liverpool, Neston, United Kingdom; ^2^Department of Musculoskeletal and Ageing Science, Institute of Lifecourse and Medical Sciences, University of Liverpool, Liverpool, United Kingdom; ^3^Department of Clinical Science and Services, Royal Veterinary College, University of London, London, United Kingdom

**Keywords:** canine, MUO, MRI, outcome, prognosis, relapse

## Abstract

**Introduction:**

Canine meningoencephalitis of unknown origin (MUO) is a debilitating disease associated with high mortality. The prognostic value of magnetic resonance imaging (MRI) findings for predicting survival at 12 months and long-term relapse remains uncertain.

**Methods:**

This was a retrospective cohort study evaluating the prognostic value of different MRI variables using multivariable logistic regression and Cox proportional hazards analysis.

**Results:**

In total, 138 dogs were presumptively diagnosed with MUO. The most common location for lesions identified on MRI were the white matter tracts of the corona radiata and corpus callosum, followed by the frontal, sensorimotor and temporal cortices. Lower T2 lesion load (*p* = 0.006, OR = 0.942, CI = 0.902–0.983) was associated with longer survival and higher T1 post-contrast lesion load (*p* = 0.023, OR = 1.162, CI = 1.021–1.322) was associated with relapse.

**Discussion:**

This study has identified prognostic factors that may help identify dogs at higher risk of death and relapse and therefore guide treatment recommendations.

## 1 Introduction

Canine meningoencephalitis of unknown origin (MUO) is a collective term used to describe a heterogenous disorder that includes clinically indistinguishable autoimmune diseases of the CNS (1–4). It comprises a group of idiopathic inflammatory diseases that can only be distinguished and confirmed on histopathology including granulomatous meningoencephalomyelitis (GME), necrotizing meningoencephalomyelitis (NME) and necrotizing leucoencephalitis (NLE). Recently, overlapping phenotypes of all these diseases has been reported, suggesting they may be different manifestations of the same disease and there is little to gain in trying to define them into different subtypes (5).

Canine MUO is a debilitating disease and despite initiation of disease-modifying treatment, 25–33% (6, 7) of dogs die within 1 week of diagnosis and 44.5% within 1-year of diagnosis (8). In view of the severity of these conditions, which are considered fatal without initiation of treatment, several studies have attempted to identify prognostic factors associated with outcome. Factors that have been associated with an improved survival include younger age (9) and early diagnosis (within 7 days of the onset of the clinical signs) (10). Conversely, seizures (7, 11–14) and mentation changes (7, 12, 15) have been associated with a worse prognosis. Recently, a neurodisability scale (NDS) has been shown to be a reliable clinical assessment tool for dogs with MUO. Initial data from a small population of dogs was unable to show an association between this score and outcome (16) but retrospective use of this scale in a large population of dogs with MUO showed an association between higher NDS score and death at 6 months as well as long-term relapse (13).

Magnetic resonance imaging (MRI) is central to the diagnosis of MUO in dogs (2, 17) so identification of MRI features associated with outcome would therefore be very useful. To date, mass effect, loss of identifiable cerebral sulci (effacement) and foramen magnum herniation were associated with increased risk of mortality but not with long-term outcome (6, 18). In human patients with multiple sclerosis (MS), several MRI findings have been associated with long-term outcome and relapse including lesion load (19–21), lesion distribution (22, 23) and brain atrophy (19, 20, 24). Due to the likely similarities between MS and MUO (1, 25) it can be hypothesized that similar variables may show prognostic value in canine MUO. As treatment efficacy may be improved by identifying specific MUO subpopulations at high risk, the aim of this study was therefore to evaluate the prognostic value of different MRI variables for predicting survival at 12 months and long-term relapse.

## 2 Materials and methods

The medical records of dogs diagnosed with MUO at the Small Animal Teaching Hospital (SATH) of the University of Liverpool and the Royal Veterinary College (RVC) were retrospectively reviewed between 2016 and 2022. Ethical approval for use of data was granted by the Ethics Committee of the University of Liverpool (VREC840). Dogs meeting the following criteria were included in the study: (1) clinical examination consistent with focal or multifocal intracranial neuroanatomical localization; (2) older than 6 months of age; (3) multiple, single or diffuse intra-axial hyperintensities on T2-weighted magnetic resonance imaging (MRI) and (4) mononuclear pleocytosis [total nucleated cell count (TNCC) > 5/μl] on cerebrospinal fluid (CSF) analysis. Dogs were excluded if no pleocytosis was found on CSF analysis, with the exception of dogs with signs of raised intracranial pressure (ICP) on imaging studies (namely transtentorial or foramen magnum herniation), for which case CSF collection was not performed. Dogs were also excluded if follow-up time was less than 12 months (except for those dogs that died during this period) or if MRI studies were incomplete.

The following data were extracted from the medical records: age, sex, neuter status, breed and duration of the clinical signs and survival information. The findings of the neurological examination recorded on the patient files were used to retrospectively assign the neurodisability scale (NDS) (16) score for each dog (calculated by the same assessor in all dogs).

MRI examinations of the head were performed using a 1.5T (Philips Ingenia CX, Philips Healthcare) scanner. The following sequences were obtained in all patients: T2-weighted images (T2W), fluid-attenuated inversion recovery (FLAIR) and pre- and post-contrast (intravenous injection of 0.1mmol/kg of gadopentetate dimeglumine) T1-weighted images (T1W).

The following MRI characteristics were recorded for each dog: location of lesions, number of lesions (focal or multifocal), contrast enhancement (present or absent), mass effect (present or absent), loss of cerebral sulci (present or absent), transtentorial herniation (present or absent) and foramen magnum herniation (present or absent). The brain was divided into three major brain zones, which were further divided into 15 brain regions. The three major brain zones were designated superficial forebrain, deep forebrain and caudal fossa. The superficial zone included the frontal, sensorimotor, parietal, temporal and occipital cortices, the piriform lobe as well as the corona radiata and corpus callosum. The deep zone included thalami, basal nuclei and internal capsule. The caudal fossa included the brainstem (midbrain, pons and medulla oblongata) and cerebellum. Lesions were assigned to a single major zone; if a lesion crossed into multiple regions, then it was assigned to the region containing the majority its volume but all zones affected were recorded.

**Figure 1:**
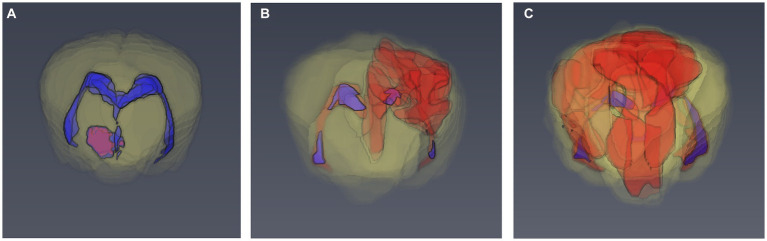
3D models of the brain of dogs based on manual segmentation of the MRI images. **(A)** 6 month old Basset Hound with 3.3% T2 lesion load. **(B)** 8 month old pug with 7.9% T2 lesion load. **(C)** 4 year old pug with 28.4% T2 lesion load.

Brain lesions were identified on T1, T2, FLAIR, and T1 post-contrast images and the total lesion volume for each sequence was calculated by one observer (RG) who was blinded to the patient details. Image processing for volume rendering was completed using graphical software (AMIRA 6.2, Thermo Fischer Scientific, UK). Segmentation techniques used were similar to previous studies in dogs (26, 27). All lesions were measured on transverse images from individual slices by free-hand measurements (Figure 1). The total brain volume and the total ventricular volume were measured on transverse T2W images. The total parenchymal brain volume was then calculated by subtracting the total ventricular volume from the total brain volume. The lesion load for each MR sequence was calculated as the ratio between total lesion volume for each sequence (T1, T2, FLAIR, and T1 post-contrast) and the total parenchymal brain volume. Precision of the volume calculation method was evaluated by calculating the intra-observer variability of the MRI measurements. This was performed by repeating the measurements in 10 randomly selected dogs by the same observer 4 weeks after the initial assessment.

Statistical analysis was undertaken using a standard statistical software package (IBM SPSS Statistics for Mac, version 29, IBM Corp, Armonk, New York). Continuous data were assessed for normality using the Shapiro–Wilk test. Descriptive statistics are reported for continuous variables using mean (standard deviation) for approximately normally distributed variables, median (interquartile range; IQR) for variables with skewed distributions, and frequencies (with 95% confidence intervals [CI] where appropriate) are reported for categorical variables. Intra-observer reliability was determined using the intraclass correlation coefficient (ICC) estimates and their 95% confident intervals (CI); these were calculated based on absolute agreement, two-way mixed-effects models. The Spearman’s rank correlation coefficient was calculated to assess the correlation between lesion load on T1, T2, FLAIR, and T1 post-contrast sequences and the NDS score, age and duration of the clinical signs. The Mann–Whitney test was used to assess the association between lesion load on T1, T2, FLAIR, and T1 post-contrast sequences and sex and resolution of the clinical signs.

Univariable binary logistic regression was performed to identify MRI variables associated with survival at 12 months after diagnosis (using the information from last examination or telephone conversation). Before multivariable analysis, all variables were assessed for correlation using Spearman’s rank correlation coefficients. If Spearman’s rank correlation coefficient was >0.8, the variable with the lowest *p*-value or most biologically plausible variable was selected. Any independent variable demonstrating some association on preliminary univariable analysis (*p* < 0.2) was considered for inclusion in a multivariable model. Multivariable models were constructed with a manual backwards stepwise removal approach; variables with *p* < 0.05 were retained. Cox proportional hazards analysis was used to identify MRI variables associated with long-term relapse using the same approach.

## 3 Results

**Figure 2:**
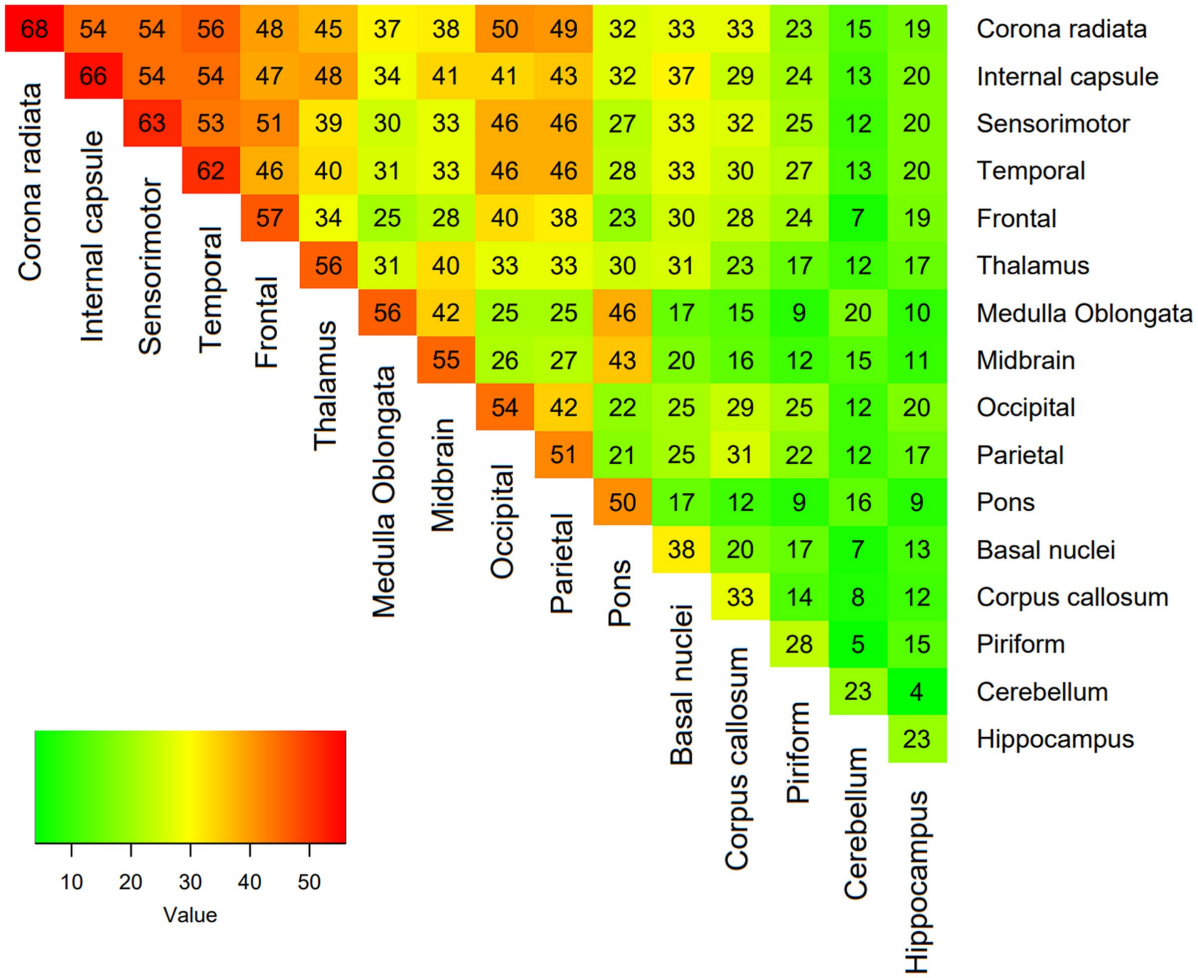
Heatmap showing the proportion of coexisting lesions at each location, with each expressed as a percentage of total lesions.

A total of 138 dogs met the inclusion criteria. The median age at time of presentation was 51 months (IQR 25–73). There were 71 (51.4%%) females (44 neutered) and 67 (48.6%) males (27 neutered). There was no association between age or sex and lesion load on MRI. The most frequently affected breeds after crossbreeds (*n* = 20) were the Pug and Chihuahua (*n* = 14 each), Maltese terrier and West Highland white terrier (*n* = 12 each), French bulldog (*n* = 10), Boston terrier and Labrador retriever (*n* = 6 each), Jack Russell terrier and Pomeranian (*n* = 4 each), Lhasa Apso, miniature Schnauzer, Yorkshire terrier, English springer spaniel and Cavalier King Charles spaniel (*n* = 3 each), Welsh springer spaniel and Shih Tzu (*n* = 2 each) and one each of 19 other breeds. Median duration of clinical signs prior to imaging was 7 days (IQR 3–14) and median NDS score on presentation was 7 (IQR 5–9). There was no association between lesion load in any sequence and duration of clinical signs, but associations between a higher NDS score and higher T2 lesion load (*p* = 0.003), FLAIR lesion load (*p* = 0.003) and T1 lesion load (*p* < 0.001) were identified. The MRI characteristics of the 138 dogs is summarized in Table 1. The most common location for lesions identified on MRI were the white matter tracts of the corona radiata and corpus callosum, followed by the frontal, sensorimotor and temporal cortices (Table 2). Distribution analysis of the lesions revealed that certain lesion locations were commonly associated, especially those that are contiguous (Figure 2).

**Table 1:** Magnetic resonance imaging (MRI) characteristics of 138 dogs diagnosed with meningoencephalitis (MUO) of unknown origin.

MRI characteristics	Dogs with MUO (*n* = 138)
**Number of lesions**	
Focal	*n* = 48 (34.8%)
Multifocal	*n* = 90 (65.2%)
**Lesion location (major zone affected)**	
Superficial zone	*n* = 68 (49.3%)
Deep zone	*n* = 14 (10.1%)
Caudal fossa zone	*n* = 56 (40.6%)
**Number of major zones affected**	
1 zone	*n* = 56 (40.6%)
2 zones	*n* = 44 (31.9%)
3 zones	*n* = 38 (27.5%)
Mass effect	*n* = 16 (11.6%)
Loss of cerebral sulci	*n* = 52 (37.7%)
Transtentorial herniation	*n* = 33 (23.9%)
Foramen magnum herniation	*n* = 23 (16.7%)
Contrast enhancing parenchymal lesions	*n* = 102 (73.9%)
Diffuse meningeal contrast enhancement	*n* = 62 (44.9%)
Median total brain volume (IQR)	73.11 cm^3^ (62.22–86.8)
Median total ventricular volume (IQR)	24.34 cm^3^ (13.52–44.97)
Median total parenchymal brain volume (IQR)	69.61cm^3^ (58.65–84.1)
Median T2 lesion load (IQR)	7.77% (3.95–15.72%)
Median FLAIR lesion load (IQR)	7.96% (3.93–15.12%)
Median T1 lesion load (IQR)	1.15% (0.3–3.34%)
Median T1 post contrast lesion load (IQR)	0.6% (0–1.75%)

FLAIR, fluid-attenuated inversion recovery; IQR, interquartile range.

**Table 2:** Location of lesions identified on magnetic resonance imaging of 138 dogs diagnosed with meningoencephalitis of unknown origin.

Location	Frequency	Percentage
Corona radiata	94	68.1
Internal capsule	91	65.9
Sensorimotor cortex	87	63
Temporal cortex	86	62.3
Frontal cortex	79	57.2
Thalamus	77	55.8
Medulla oblongata	77	55.8
Midbrain	76	55.1
Occipital cortex	74	53.6
Parietal cortex	71	51.4
Pons	69	50
Basal nuclei	53	38.4
Corpus callosum	46	33.3
Piriform lobe	37	26.8
Cerebellum	32	23.2
Hippocampus	32	23.2

There was excellent intra-observer reliability (28) for the measurements in all sequences: T2 lesion load (ICC 0.999, 95% CI 0.997–1), FLAIR lesion load (ICC 0.996, 95% CI 0.985–0.999), T1 lesion load (ICC 0.962, 95% CI 0.856–0.99) and T1 post contrast lesion load (ICC 0.980, 95% CI 0.921–0.995).

All dogs received immunosuppressive doses of dexamethasone at the time of diagnosis alongside cytosine arabinoside in 84.6% (111/130) of dogs (subcutaneous injection in 39.6% and constant rate infusion in 60.4%). Eight dogs died shortly after diagnostic investigations (did not recover from anesthesia) so did not receive any treatment other than corticosteroids and in 11 dogs, owners refused treatment with cytosine arabinoside at the time of diagnosis. Immunomodulatory drugs used long term varied substantially between patients in terms of dose and length of treatment but included prednisolone (101), cytosine arabinoside administration every 3–8 weeks (65), cyclosporine (18), leflunomide (12), azathioprine (4), and procarbazine (2).

Median follow-up time was 10 months (IQR 0.2–20.5). Seventy-five percent (101/138) of dogs survived to discharge and 47.8% (66/138) were alive at 12 months. Complete resolution of the clinical signs was not seen in 30.7% (31/101) of dogs during the follow-up time, despite clinical improvement. Lack of resolution of the clinical signs was not associated with lesion load on any MR sequence.

The Spearman’s rank correlation coefficient between T2 and FLAIR lesion load was 0.951 so only T2 lesion load was included in the logistic regression model. On univariable logistic regression, T2 and T1 lesion load, loss of cerebral sulci, and foramen magnum herniation showed some evidence of association with survival at 12-months (Supplementary Table S1). However, on multivariable analysis, only a smaller T2 lesion load (*p* = 0.006, OR = 0.942, CI = 0.902–0.983) was associated with higher likelihood of survival at 12 months.

Relapse occurred in 57.5% (57/101) of dogs that survived to discharge (median time to relapse 8.5 months, IQR 3–18). On univariable Cox regression analysis, only presence of contrast enhancement, T1 and T1 post-contrast lesion load showed some evidence of association with relapse (Supplementary Table S2). On multivariable analysis, only higher T1 post-contrast lesion load (*p* = 0.023, OR = 1.162, CI = 1.021–1.322) was associated with relapse.

## 4 Discussion

This is the largest study to date evaluating MRI prognostic factors for survival and relapse in MUO. We have identified that a lower T2 lesion load was associated with survival at 12 months and that a higher T1 post contrast lesion load was associated with relapse. We have also identified an association between higher NDS score and higher T2, FLAIR, and T1 lesion load. The most commonly affected major zone of the brain was the superficial zone, with the white matter in the corona radiata and internal capsule as the most commonly affected regions.

Previous studies had identified that mass effect, loss of identifiable cerebral sulci and foramen magnum herniation were significantly associated with increased risk of mortality (6, 18), but this had not been reproduced in other studies (7, 9, 15). In our population, despite the reasonably high frequency of these abnormalities, we could not replicate these findings.

We have nonetheless identified an association between T2 lesion load and survival at 12 months after diagnosis, even if with a modest effect. T2 lesion load had been previously associated with longer time from disease onset but not with outcome in pug dogs with NME (29). Median survival time of the 18 dogs included in that study was 18 days only and this likely would make interpretation of these results difficult. Similar results to ours have been found in humans with MS, where T2 lesion loads on baseline MRI are associated with worse outcomes (19, 20, 30, 31). This association has been reported to be modest in some studies but in those with longer follow-up durations, stronger associations are identified (32, 33). It can be speculated that similar findings may be identified in dogs with MUO and future studies with longer follow-up periods are warranted.

We have found that a higher NDS score was associated with higher lesion load on T2, FLAIR and T1. A previous study identified that a higher NDS score was associated with increased risk of death within the 6-months after diagnosis and incomplete resolution of the clinical signs within that time as well as long-term relapse (13). In human medicine, the combination of MRI and clinical markers has shown to increase prediction of disability in MS, allowing generation of a composite predictive score based on the best individual predictors (19). These included T2 lesion load and the Expanded Disability Status Scale (EDSS) score among other variables and it is likely that a similar approach combining clinical and MRI findings may also prove beneficial in dogs.

Contrast enhancement was present in approximately three quarters of dogs with MUO. The presence or absence of contrast enhancement itself was not associated with survival or relapse but higher T1 post-contrast lesion load was associated with risk of relapse. A moderate positive association between parenchymal contrast enhancement and both necrosis and monocytic inflammation in pug dogs with NME has been previously reported (29). The uptake of intravenously administered contrast material by brain tissue signals blood–brain barrier (BBB) breakdown (34) and mononuclear cell infiltrates (35). In human patients with MS, it has been shown that new inflammatory lesions take up gadolinium for only around 3 weeks (36). Our findings echo those in human patients with MS where gadolinium enhancement is associated with relapse but not long-term disability (35).

The most commonly affected major zone of the brain was the superficial zone and the least affected was the deep zone. The white matter in the corona radiata and internal capsule were the most commonly affected regions followed by the frontal, sensorimotor and temporal cortices. No associations between the major zone affected and survival or relapse were identified. This is different to in human patients with MS, where lesions located in specific brain tracts (corpus callosum, corona radiata and cingulum) are associated with a worse outcome (37). Also, the presence of intratentorial T2 lesions has been associated with worse prognosis (38). Approximately two thirds of the dogs in this study presented multifocal lesions on MRI. One previous study had identified that multifocal neurolocalization was associated with a worse prognosis than focal neurological deficits (39) but subsequent studies have failed to reproduce these findings (6, 12, 15). Our study evaluated the possible prognostic value of focal versus multifocal lesions on MRI, rather than on the neurological examination findings, but also failed to identify any association with outcome.

Aside from the invaluable role in the diagnosis of MS, MRI is now established as a central part of the routine medical management of individual patients with this disease (40). In clinical practice, serial MRI is routinely used to assess patients during follow-up, guide treatment decisions and crucially predict the course of disease (40, 41). In dogs with MUO, resolution of abnormalities on repeat MRI and on CSF analysis at 3 months after diagnosis was associated with a good outcome (6). As there is still much controversy regarding the gold standard treatment for dogs with MUO (1), deciding on the most suited initial treatment based on identification of MRI variables associated with worse prognosis in dogs remains challenging. It could nonetheless alert clinicians to the possible benefit of more frequent re-examinations or repeat MRI to monitor response to treatment. Earlier detection of new lesions or incomplete resolution of baseline lesions could guide faster changes to the treatment protocol (either increased doses of previously used medications or introduction of new medications) and possibly prevent more severe clinical relapse.

There were several limitations in this study. The retrospective nature of the study means that the treatment received was not completely standardized and it is likely that different treatment protocols will affect prognosis. Unfortunately, large prospective, blinded randomized clinical trials comparing the different treatments directly to each other are not available and this results in a large variation of treatment protocols used. Nonetheless, our data reflects common clinical practice and the results could therefore be applicable in a clinical setting. Due to the inclusion of several dog breeds with large variability of head and brain size, lesion burden was calculated as a percentage of brain volume. The ventricular volume was initially subtracted from the total brain volume to calculate the total parenchymal volume. This approach may result in underestimation of the lesion volume in dogs with ventriculomegaly secondary to brain atrophy or overestimation in dogs with ventriculomegaly secondary to head conformation. Most lesions identified on MRI were ill-defined and contrast enhancement was in many cases patchy or linear, making delineation of the lesions less clear in some cases; this did not seem to significantly affect the results as intra-observer reliability for the measurements was excellent in all sequences. Lastly, there was no histopathological examination in most cases so it is possible that patients had diseases other than MUO or that some of the lesions measured were secondary to peri-ictal changes rather than inflammatory lesions.

This study has identified that lower T2 lesion load was associated with survival and higher T1 post contrast lesion load was associated with relapse. Knowledge of these MRI prognostic factors may help identify dogs at higher risk of death and relapse and therefore guide treatment recommendations accordingly. These could include more frequent re-examinations or advice to repeat diagnostic investigations during treatment to monitor the baseline changes identified.

## Data availability statement

The datasets presented in this article are not readily available because the MR images have identifiable pet owner data. Requests to access the datasets should be directed to ritag@liverpool.ac.uk.

## Author contributions

RG: Conceptualization, Data curation, Formal analysis, Investigation, Methodology, Project administration, Software, Validation, Writing – original draft, Writing – review & editing. SD: Data curation, Investigation, Writing – original draft, Writing – review & editing. GW: Conceptualization, Investigation, Methodology, Supervision, Writing – original draft, Writing – review & editing. TM: Conceptualization, Formal analysis, Investigation, Methodology, Supervision, Writing – original draft, Writing – review & editing.
