# Corrigendum: Magnetic resonance imaging prognostic factors for survival and relapse in dogs with meningoencephalitis of unknown origin

**DOI:** 10.3389/fvets.2024.1456528

**Published:** 2024-07-23

**Authors:** Rita Gonçalves, Steven De Decker, Gemma Walmsley, Thomas W. Maddox

**Affiliations:** ^1^Department of Veterinary Science, Small Animal Teaching Hospital, University of Liverpool, Neston, United Kingdom; ^2^Department of Musculoskeletal and Ageing Science, Institute of Lifecourse and Medical Sciences, University of Liverpool, Liverpool, United Kingdom; ^3^Department of Clinical Science and Services, Royal Veterinary College, University of London, London, United Kingdom

**Keywords:** canine, MUO, MRI, outcome, prognosis, relapse

In the published article, there was an error.

The authors omitted acknowledging the support from a different department within the university, which breaches the University of Liverpool's guidelines for interdepartmental collaborations. In order to provide this information, the Acknowledgments section has been added.

The corrected sentence appears below:

