# Supplementary material for: Magnetic resonance imaging prognostic factors for survival and relapse in dogs with meningoencephalitis of unknown origin
Source: Front Vet Sci. 2024 Feb 28;11:1370882. doi: 10.3389/fvets.2024.1370882 (PMC10933066; doi:10.3389/fvets.2024.1370882)
Supplement: Supplementary file 1 [file Table_1.DOCX]

Supplementary tables

Supplementary table 1. Univariable logistic regression analysis results evaluation associations between different MRI variables associated and survival at 12 months after diagnosis. Variables with an asterisk were subsequently used in the multivariable model.

| MRI Characteristics | OR | 95% CI | *P*-value |
| --- | --- | --- | --- |
| Number of lesions (focal vs multifocal) | 0.994 | 0.493-2.005 | 0.988 |
| Lesion location (major zone affected)  Superficial zone  Deep zone  Caudal fossa zone | ref  0.950  1.462 | ref  0.297-3.036  0.718-2.975 | ref  0.931  0.295 |
| Mass effect | 1.103 | 0.389-3.129 | 0.853 |
| Loss of cerebral sulci* | 0.478 | 0.236-0.968 | 0.04 |
| Transtentorial herniation | 0.751 | 0.341-1.645 | 0.477 |
| Foramen magnum herniation* | 0.415 | 0.159-1.085 | 0.073 |
| Contrast enhancing parenchymal lesions | 1.202 | 0.56-2.579 | 0.637 |
| T2 lesion load* | 0.942 | 0.902-0.983 | 0.006 |
| T1 lesion load* | 0.805 | 0.695-0.934 | 0.004 |
| T1 post contrast lesion load | 0.935 | 0.771-1.133 | 0.491 |

CI – confidence interval; OR – odds ratio

Supplementary table 2. Cox proportional hazards analysis results evaluating association between MRI variables and long-term relapse. Variables with an asterisk were subsequently used in the multivariable model.

| MRI Characteristics | OR | 95% CI | *P*-value |
| --- | --- | --- | --- |
| Number of lesions (focal vs multifocal) | 1.068 | 0.607-1.879 | 0.820 |
| Lesion location (major zone affected)  Superficial zone  Deep zone  Caudal fossa zone | ref  1.553  1.085 | ref  0.668-3.609  0.615-1.917 | ref  0.306  0.778 |
| Mass effect | 0.684 | 0.272-1.72 | 0.419 |
| Loss of cerebral sulci | 1.201 | 0.683-2.114 | 0.525 |
| Transtentorial herniation | 1.083 | 0.542-2.165 | 0.821 |
| Foramen magnum herniation | 1.751 | 0.781-3.925 | 0.205 |
| Contrast enhancing parenchymal lesions* | 1.838 | 0.923-3.659 | 0.083 |
| T2 lesion load | 1.021 | 0.989-1.054 | 0.201 |
| T1 lesion load* | 1.108 | 0.986-1.245 | 0.085 |
| T1 post contrast lesion load* | 1.162 | 1.021-1.322 | 0.023 |

CI – confidence interval; OR – odds ratio
